# Functionalization of Synthetic Bone Substitutes

**DOI:** 10.3390/ijms22094412

**Published:** 2021-04-23

**Authors:** André Busch, Marcus Jäger, Constantin Mayer, Andrea Sowislok

**Affiliations:** 1 Department of Orthopedics, Trauma and Reconstructive Surgery, St. Marien Hospital Mülheim an der Ruhr, D-45468 Mülheim, Germany; an.busch@contilia.de (A.B.); c.mayer@contilia.de (C.M.); 2 Department of Orthopedics and Trauma Surgery, University Hospital Essen, University of Duisburg-Essen, D-45147 Essen, Germany; andrea.sowislok@uni-due.de; 3 Chair of Orthopedics and Trauma Surgery, University of Duisburg Essen, D-45147 Essen, Germany

**Keywords:** BMP, tissue engineering, implant related infections, antibiotics

## Abstract

Bone substitutes have been applied to treat osseous defects for a long time. To prevent implant related infection (IRI) and enhance bone healing functionalized biomaterials, antibiotics and osteoinductive substances have been introduced. This study gives an overview of the current available surface-coated bone substitutes and provides an outlook for future perspectives.

## 1. Introduction

Bone is a highly active tissue with high functional stability and regenerability [1]. However, in some cases bone defects due to trauma, tumor or infection the self-healing capacity of the bone tissue is impeded [2]. Autologous bone transplantation remains the gold standard to treat bone defects. Though, donor site morbidity and finite resources limit the applicability of autologous bone tissue [3]. The application of allogenic bone materials is restricted to high rates of hard and soft-tissue infections and contamination with osteotoxic substances [4,5]. Therefore, major efforts have been made to create alternatives for treatment of bone defects. The requirements on synthetic bone substitutes include osteoconduction, osteopromotion and osteoinduction [6]. Osteoconductive materials (ß-TCP; HA, etc.) function as scaffolds allowing osteoblast precursor cell ingrowth on the outer and inner surface leading to new bone formation [7]. Osteopromotion is the physical stimulus to boost bone formation in bone healing [8]. Osteoinductive materials are able to stimulate bone formation even in a non-osseous environment by recruiting undifferentiated and pluripotent progenitor cells [7].

The current trend is going to combine those properties of different materials to enhance bone-healing processes [9]. Another trend emerging is to supplement bone substitute materials with antibiotics to prevent and treat bone infections [10].

## 2. Overview of Bone Fracture Healing

Modern tissue engineering approaches for bone regeneration are inspired by natural bone healing, in which bone formation and regeneration is controlled by a complex interplay of molecules delivered at precise locations at a defined time [11]. The key processes of fracture healing can be summarized by the release of cytokines as a result of inflammation triggering the subsequent formation of a cartilaginous callus, which later undergoes mineralization, resorption and replacement with bone.

After a fracture, the breach in the structural continuity of the bone cortex and the destruction of soft tissue and blood vessels supplying the bone and the periosteum lead to necrosis, inflammation and hematoma around the fracture site [12]. The coagulated hematoma in between and around the fracture ends serves as a temporary template for callus formation by stem cell differentiation into fibrous tissue and cartilage [13]. Furthermore, pro-inflammatory cytokines like tumor necrosis factor-alpha (TNF-α), bone morphogenetic proteins (BMPs), vascular endothelial growth factor (VEGF) and interleukins (IL-1, IL-6, IL-11, IL-23) are released in the first phase of fracture healing by macrophages and other inflammatory cells [14]. This stimulates blood flow, angiogenesis and chemotactic recruitment of necessary cells like mesenchymal stem cells (MSCs), macrophages, monocytes and lymphocytes, and the triggering of mononuclear phagocytes to reabsorb necrotic bone fragments [15]. The second phase of bone healing is marked by the formation of a soft fibrocartilaginous callus giving the fracture a stable structure [16]. The preliminary hematoma transitions into a fibrin-rich granulation tissue. Angiogenesis and vasculogenesis at the fracture site is induced by the release of VEGF and angiopoetin -1 and -2 [17]. The release of BMPs stimulates MSCs to differentiate to chondrocytes and osteoblasts leading to chondrogenesis [18]. A collagen-rich fibrocartilaginous network is spanning the fracture ends, with a surrounding hyaline cartilage sleeve. A layer of woven bone is laid down by the osteoprogenitor cells adjacent to the periosteal layers at the same time [13]. In the third phase of fracture healing, the cartilaginous callus undergoes endochondral ossification, where the initial soft callus is resorbed and replaced by a hard bony callus. Subperiostally, woven bone continues to be laid down [19]. As fracture callus chondrocytes proliferate, they become hypertrophic and the extracellular matrix is calcified under the secretion of alkaline phosphatase, which acts as a nucleator for the deposition of minerals on the template [20]. In chondrocyte mitochondria, calcium-containing granules are created in the hypoxic fracture environment. After the release of these granules to the cytoplasm, they are transported into the extracellular matrix where they precipitate with phosphate and form initial mineral deposits. The deposition of calcium and phosphate becomes the nucleation site for the formation of apatite crystals [21]. The release of macrophage colony-stimulating factor (M-CSF), receptor activator of nuclear factor kappa B ligand (RANKL), osteoprotegerin (OPG) and TNF-α initiate the resorption of this mineralized cartilage under chondrocyte apoptosis and subsequent removal by chondroclasts [22]. The vascularization of the callus proceeds and woven bone tissue is laid down as blood vessels from the perichondrium penetrate the remaining cavities and recruit hemopoietic cells and osteoprogenitor cells [13]. The resulting hard callus is a rigid structure providing biomechanical stability, but it still needs to undergo a remodeling phase in order to gain the fully restored biomechanical properties of normal bone [20]. This fourth and last phase of fracture healing is biochemically dependent on IL-1 and TNF-α and is marked by the balanced resorption of hard callus by osteoclasts and the deposition of lamellar bone with a central medullary cavity by osteoblasts [23]. Weight-bearing stresses induce bone remodeling by creating an electrical polarity as a result of an applied pressure in a crystalline environment. When axial loading of long bones occur, convex surfaces get an electropositive charge while concave surfaces get electronegative. According to Wolff’s law, osteoblastic activity is enhanced on electronegative surfaces and osteoclast activity is higher on electropositive surfaces. This results in a gradual replacement of the external callus by a lamellar bone structure, while the internal callus remodeling leads to a medullar cavity being characteristic for a diaphyseal bone [13].

## 3. First Generation of Growth Factor Delivering Biomaterials

One of the most potent osteoinductive growth factors are bone morphogenetic proteins (BMPs) [11]. These multifunctional cytokines are involved in all different stages of fracture healing by inducing the differentiation of MSCs into chondrogenic and osteogenic lineages, stimulating angiogenesis and increasing alkaline phosphatase activity [24]. BMPs are non-collagenous glycoproteins of low molecular weight belonging to the transforming growth factor-beta TGF-β superfamily [25]. They are highly conserved proteins with covalently disulfide-linked dimeric structures [26]. Even though over 20 homodimeric or heterodimeric BMPs have been identified, only a few members of this family, such as BMP-2,-3,-6,-7 and 9, are truly osteogenic [24]. The osteogenic potential of BMPs was first discovered by Urist in 1965 when the induction of bone formation was shown after the implantation of demineralized bone matrix into ectopic sites in rats [27]. In the past, the access to BMPs was limited due to their low yield after isolation from bovine cadaver bone [28]. The milestone for the commercially availability of BMPs was reached in 1988, when Wozney first cloned Recombinant human BMP-2 and expressed it in chinese hamster ovary cells [29]. Nowadays, all clinically available rhBMPs are derived from mammalian cell cultures transfected by the BMP-gene [26].

Until today, only BMP-2 (Infuse^®^ bone graft) and BMP-7 (OP-1 putty^®^), which is also known as osteogenic protein 1 (OP-1), have an FDA approval for treating very specific bone fractures that exhibit delayed or incomplete healing. However, OP-1 putty was withdrawn from the market in 2014 [11]. The remaining product Infuse is costly and requires supraphysiologic concentrations (10–1000 fold higher) to induce bone healing. Both bone grafts use carrier systems based on collagen type 1: Infuse utilizes absorbable collagen sponges (ACS) and putty collagen particles [11]. The use of a natural component like collagen for this purpose seemed to be very promising due to its biodegradability, biocompatibility and its ability to support mineralization and cell ingrowth in an osteoconductive manner [30]. However, there are several severe side effects in clinical applications associated with this carrier system [28]. The positioning of the ACS during surgery is often difficult and in some cases, secondary displacement of the collagen sponge takes place [31]. The osteoconductive properties of collagen matrices is limited due to its quick degradation in vivo, leading to an insufficient structure half-life able to support cell migration [32]. The main issue associated with the ACS is an initial burst release of BMP-2 into the local environment due to the low binding affinity of BMPs towards collagen [33]. This might induce heterotrophic ossification in muscle tissue as surrounding mesenchymal lineage progenitor cells in adjacent musculature differentiate into osteoblasts and cause mineral deposition under BMP stimulation [34]. High BMP-2 concentrations might also activate osteoclasts, leading to bone resorption [35]. Further side effects are inflammation, bone cysts and neurological impairment with regard to spine surgery [36].

Therefore, there is a considerable need for providing a suitable delivery system or vehicle for the controlled and continuous release of BMPs.

## 4. Alternative Carriers for Growth Factor Delivery

The biggest challenge when developing osteoinductive bone grafts is the short systemic half-life of growth factors like BMP in the bloodstream characterized by their rapid degradation by proteinases after only 7 to 16 min [36]. Supraphysiologic BMP concentrations in clinical applications try to compensate the short retention time at the defect site in order to enhance signaling efficiency, but lead to severe side effects due to a burst release [36]. Therefore, there is a need for new biomaterials that allow spatiotemporal sustained release of growth factors, so that a prolonged presence of BMPs at the implantation site is guaranteed, their systemic diffusion is avoided and their local concentrations is kept on a constant physiological level [24]. The requirements for the ideal growth factor carrier are enormous and until today, no carrier really meets all expectations. An osteoconductive three-dimensional highly porous carrier structure should present adhesion sites for cell ligands, contain affinity motifs for growth factor binding, fill in the defect and should have appropriate mechanical properties. It should be biocompatible and biodegradable, while protecting BMPs from degradation. Furthermore, it should be non-toxic, non-allergic, non-carcinogenic, easily sterilized, stable and cost-effective [37].

Several natural and synthetic polymers have been further investigated as possible carrier materials for bone tissue engineering in the past years [38].

Natural polymers like collagen, fibrin, chitosan, hyaluronic acid, gelatin and alginate have distinct advantages due to their inherent biocompatibility and bioactivity, but lack the mechanical properties required for load bearing applications. Furthermore, they have fixed degradation rates, are difficult to harvest and sterilize and exhibit batch-to-batch variability. In some cases, they have a risk of pathogen transmission and may induce an immunogenic response [39].

Synthetic polymers have a defined chemistry, they are tunable with regard to porosity and degradation time, but they lack inherent bioactivity. They can be produced in large quantities under controlled conditions, have a long shelf life, are easy to process and often cheaper than biological scaffolds [40]. The most commonly used synthetic polymers in tissue engineering applications with a FDA approval clinical applications are aliphatic polymers like polylactic acid (PLA), polyglycolic acid (PGA), polylactic-co-glycolide (PLGA), poly(ɛ-caprolactone) (PCL), poly-p-dioxanone and copolymers consisting of glycolide/trimethylene carbonate [40].

Besides all different materials for tissue engineering purposes, different strategies for the synthesis of novel carrier systems have evolved. Synthetic bone grafts can be synthesized through solvent casting and particulate leaching (SCPL), freeze-drying, thermally induced phase separation (TIPS), gas foaming, electrospinning, hydrogel formation and additive manufacturing [41].

Since the function of many proteins is highly dependent on their structure, the loading of growth factors to a scaffold represents a critical step in functional biomaterial development [42]. The native protein conformation and their bioactivity should be preserved throughout scaffold loading and during their release in vivo [43]. The use of organic solvents for the creation of BMP-loaded scaffolds can impair its bioactivity by affecting the quaternary and tertiary protein conformation [44]. Additionally, secondary protein-polymer interactions can trigger protein misfolding and aggregation. Degradation products of polymeric scaffold can increase the local acidity and lead to protein denaturation or degradation [45].

Over the years, many different strategies for the incorporation of growth factors into biomaterials have evolved, including physical entrapment, chemical and affinity binding and surface modifications strategies [46].

In general, the release of growth factors from a carrier is dependent on their chemistry, their physical affinity towards the carrier and on the chemical and physical nature of the scaffold itself [42]. A rapidly degrading biomaterial expresses a burst release, while biomaterials with low biodegradability have slow release profiles [47]. Dependent on the used incorporation strategy, the release of a growth factor can be controlled by either diffusion, solvent changes, chemical reactions, or a combination of these mechanisms. In cases where BMP is physically immobilized, it is released by the degradation of the carrier in a chemically controlled way. When BMP is implemented in the pores of a porous scaffold, the release is controlled by diffusion [36].

## 5. Examples of Synthetic Biomaterials for Growth Factor Delivery

**Figure 1:**
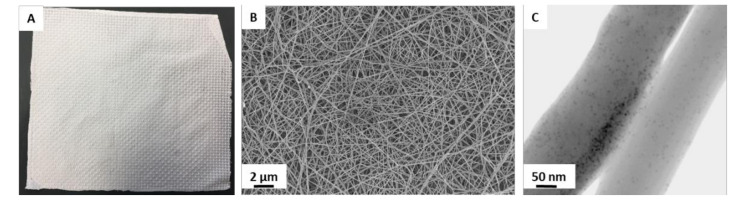
Macroscopic and microscopic pictures of a PDLLA fleece made by electro spinning as an example for a tissue-engineered biomaterial for growth factor delivery. (**A**) Macroscopic photo of a polylactide fleece made by electro spinning. (**B**) SEM image reveals typical non-woven, fibrous structure with random orientation. (**C**) TEM image shows iron containing ferritin molecules adsorbed on the fiber surface. (unpublished results from symposium presentation [54]).

As shown before, appropriate growth factor delivery is a complex interplay between the carrier material, the fabrication technique of the scaffold and the incorporation strategy of the growth factor. Based on these concepts, different researchers have synthesized several scaffolds for BMP delivery, which had been evaluated in in vitro and in vivo for their bone regenerating potential [48]. However, none of these carrier systems is in clinical trials. Modern tissue engineering approaches do not only focus on the single delivery of a certain compound, but also combine multiple bioactive molecules and multiple scaffold materials [49]. Since bone healing is highly dependent on vascularization, some biomaterials combine the delivery of growth factors for bone regeneration with angiogenic factors to stimulate vessel formation [50]. By using polymers with different degradation rates, angiogenic factors can be released prior to growth factors, which is in line with natural fracture healing [49]. For example, Kanczler et al. created a composite scaffold consisting of a porous BMP-2 containing PLA matrix with embedded alginate fibers with incorporated VEGF by using supercritical carbon dioxide [51]. The release kinetics of BMP-2 encapsulated in such foamed PDLLA scaffolds were determined with half-lives of 86–348 days depending on the temperature [52]. Besides encapsulation of BMP-2 and VEGF within nanofibers, these growth factors can also be adsorbed on the outer surface [53,54]. An example for the visualization of adsorbed proteins, e.g., of ferritin on nanofibers, is shown in Figure 1 [54].

Desorption kinetics from PDLLA fleeces showed a short burst and a sustained release phase and revealed that VEGF (t_1/2_~19 days) was released at an 11-fold higher rate than BMP (t_1/2_~209 days), indicating a lower binding affinity of VEGF for PDLLA than BMP [53]. Furthermore, in vitro bone healing models showed that both biomaterials were non-toxic and led to the activation of microvessel formation [55].

The combination of ceramic based biomaterials with polymeric carriers shows a different facet of modern tissue engineering approaches.

On the hydroxyl apatite based bone replacement material Algoss it could be shown in vitro that the desorption kinetics of adsorbed BMP-2 and VEGF from the hydroxyl apatite-based bone replacement material Algoss yielded a half-life for sustained release of 23 days for VEGF and 101 days for BMP-2, respectively [56]. As shown by Asran et al., hydroxyapatite nanoparticles (nHAp) can be incorporated into electrospun materials made of polyvinylalcohol (PVA) and collagen type I (Col) to create composite nanofibrous membranes. The obtained PVA/Col/nHAp scaffolds have a rigid and porous structure with an adjustable pore size in the range of 650 nm [57].

Dual functional tissue-engineered biomaterials can also combine osteoinductive growth factors with antimicrobial agents in order to promote bone regeneration and reduce infections simultaneously, which will be presented in more detail later in this article [50].

## 6. Antibiotic Eluting Bone Substituents

Surgery with bone substitutes is time-consuming and technically demanding [58]. The increasing number of orthopedic implants are associated with an increase of implant-related infections (IRI) representing a significant clinical, public and economic burden. The infection rates under use of bone substitute materials are reported to be up to 12% [3]. The bacterial spectrum in IRI consists with mainly staphylococci followed by streptococci, enterococci, Gram-negative rods and anaerobs [59]. This is in line with the findings in periprosthetic joint infection (PJI). Worryingly, Bjerke-Kroll et al. observed an increase of methicillin-resistant *Staphylococcus aureus* (MRSA) bacteria in PJI [60]. Furthermore, a growing body of evidence suggests high rates of PJI caused by *Cutibacterium acnes* (*Propioni-bacterium acnes*) and gram negative germs [61,62].

Surgical antimicrobial prophylaxis with a beta-lactam antimicrobial, is a cornerstone in prevention of IRI [63]. Yet, current guidelines reflect on research undertaken over 30 years ago, although there has been an increased incidence of antimicrobial resistant organisms [64]. On the surface of foreign materials, biofilms can be formed due to lack of immune response [65]. Therefore, these infections often require the removal of the device and implantation of revision implants [66].

If IRI occurred, systemically applied antibiotics cannot reach an infected implant sufficiently [67]. The penetration of systemically applied antibiotics into soft-tissue and bone is poor especially in patients with peripheral arterial occlusive disease [68]. Furthermore, the minimal inhibitory concentrations (MIC) of systemically applied antibiotics vary from organism to organism [69]. β-Lactam antibiotics (penicillins, cephalosporins, and carbapenems) penetrate bone only at levels ranging from 5% to 20% of those in serum. Parenterally applied ß-lactam antibiotics reach high serum levels exceeding MICs of etiologic bacteria in most osteomyelitis cases, whereas oral ß-lactam agents do not achieve adequate bone levels [70,71]. Similar to ß-lactam antibiotics, vancomycin poorly penetrates into bone [72]. If serum levels exceed 35 μg/mL, its penetration into bone reaches 30% of serum concentrations [73]. Yet, using vancomycin concentrations above 20 μg/mL can cause nephrotoxic effects [74].

Local application of antibiotics to prevent and treat bone infections and IRI was performed for many years [75]. Injection of aqueous antibiotic solutions is probably effective in the treatment of areas close to articulations [76]. Yet, antibiotic concentrations do not remain on high levels after injection for a long time. Therefore, aqueous antibiotic solutions may only act as prophylactic applications [77]. To reach sufficient antibiotic concentrations at the implant site, local biodegradable antibiotic-impregnated materials have been devised [78]. In contrast to non-biodegradable carriers, bone substitutes have the advantage of remodeling through endogenous tissue so that after complete release of the antibiotic the risk of an infection of the foreign material is negligible [77]. However, the release of antibiotics from bone substitutes is very fast and relatively erratic [79]. The elution of the antibiotics was demonstrated with an initial burst in the first 48 h. The antibiotic levels reach about hundreds to thousands times higher than MIC [80,81] and potentially above the mean biofilm eradication concentration [82]. However, high levels of antibiotics, such as gentamicin, achieved following topical application, were shown to inhibit cell proliferation in vitro [11]. This may lead to impaired blood vessel ingrowth and delayed bone healing [83].

Many efforts have been made trying to find solutions combining bone substitutes with antibiotics. At present, there are only a few commercially available antibiotic-loaded bone substitutes licensed for the treatment of osteomyelitis [24]. The list of available antibiotics contains only tobramycin, gentamicin and vancomycin. The major problem in combining antibiotics with bone substitutes is the exothermic polymerization reaction during material processing with fatal consequences to heat-sensitive antibiotics [84].

There is currently less information about the susceptibility of the organisms causing IRI to aforementioned antibiotics. Lawrie et al. (2020) reported on the susceptibility of organisms causing PJI in TKA (total knee arthroplasty). The gram-positive isolates were totally susceptible to vancomycin. Overall, 22% and 27.8% of the staphylococcal germs were resistant to gentamicin and tobramycin, respectively. Of the *Enteroccous faecalis* germs, 100% were resistant to aminoglycosides. The remaining gram-negative germs were universally resistant to vancomycin but susceptible to gentamicin and tobramycin [85].

The discussion about the potential resistance development caused by local antibiotics is still open. The slow penetration rate of antibiotics through the biofilm matrix allows the bacteria to adapt phenotypically and genotypically [12]. The horizontal gene transfer in sessile bacteria is 10,000 times more likely than in planktonic bacteria [86,87]. Beyond that, bacteria in a biofilm tend towards spontaneous mutations [88]. A further progression of resistance of IRI causing bacteria could have devastating consequences. The increasing rate of vancomycin-resistant enterococci (VRE) [89] should be alarming. Moreover, vancomycin intermediate resistant *Staphylococcus aureus* (VISA) and vancomycin-resistant *Staphylococcus aureus* (VRSA) are increasing [90]. As aforementioned, the rate of gram negative implant infections is underestimated. Especially infections with *Pseudomonas aeruginosa* have a particularly high treatment failure rate and oftentimes require more surgical interventions [91]. The influence of antibiotic addition on the biological behavior of bone substitutes is not fully resolved. It is known that gentamycin decelerates the hydration process of calcium sulfate. High amounts of gentamycin result in faster degradation and lower mechanical strength of calcium sulfate [92]. In contrast, doxycyclin doped deproteinized cancellous bovine bone showed statistically increased mineralized new bone formation in comparison to cancellous bovine bone alone. Furthermore, an upregulation of TGFβ1, BMP-2 and β-catenin were evident through the addition of doxycyclin [93].

The following two recently published examples use hydrogels as carrier to combine BMP-2 with vancomycin or lysostaphin, which lead to bacterial lysis and biofilm reduction [94,95]. Lysostaphin is a metallic-endopeptidase produced by *Staphylococcus simultans* with antimicrobial activity specific against staphylococcal species. Hydrogels are three-dimensional cross-linked polymer networks, which can absorb and retain large amounts of water without dissolving due to their cross-links [96]. They are nontoxic, provide a well-defined mesh structure, can be produced under mild reaction conditions and allow precise stoichiometric incorporation of biomolecules and cell-adhesive ligands [94].

Johnson et al. created an implantable hydrogel based on the synthetic polymer four-arm polyethylenglycol-maleimid (PEG-4MAL) in a one-pot reaction by mixing the protease-degradable cross-linking peptide VPM and the cell-adhesive peptide GFOGER. 1 U Lysostaphin and 100 ng BMP-2 were physically entrapped within the mesh structure and are released by diffusion and degradation of the hydrogel. In vivo studies of a nonhealing infected segmental bone defect in the murine radius showed that the biomaterial effectively eliminates *S. aureus* infection while simultaneously regenerating functional bone. At the infection site, lysostaphin returned the inflammatory environment to that of an uninfecteted defect. The mechanical properties of the regenerated bone was similar to those of intact mouse radii [94].

Jung et al. created an injectable in situ gelling hydrogel based on the natural polymers alginate and hyaluronic acid by tuning the gelation time to four minutes by adjusting the relation of the cross-linking agent CaSO_4_ and the corresponding retardation agent Na_2_HPO_4_. 1 µg/mL BMP-2 was physically entrapped within the hydrogel, while 50 mg/mL of the positively charged vancomycin was incorporated through electrostatic interactions with the negatively charged alginate. In vitro release kinetics showed a slow, sustained release of vancomycin over 6 weeks due to slow dissociation from alginate in the physiological environment. In the case of BMP-2, a burst release on the first day and a sustained release for 6 weeks was observed. The burst release is a consequence of the rapid desorption of absorbed BMP on the alginate scaffold, the slow release depends on the diffusion through the porous network of the carrier. The in vivo study of an osteomyelitis femur Sprague-Dawley rat model showed that the biomaterial was effective in suppressing *S. aureus* proliferation at the osteomyelitis lesion and that bone regeneration was enhanced [95].

These in vivo studies illustrate the tremendous potential of functional synthetic biomaterials in handling infections and bone regeneration. However, the efficacy and the reliability of these results obtained from animal studies is in doubt and is controversial when it comes to applications in human beings, since studies showed that rodents, lagomorphs and canine tissues respond differently when compared to primate tissue and its microenvironment [97].

## 7. Surgical-Site Surface Coating

The research to augment bone substitutes intraoperatively is emerging [98]. There are several established systems such as reamer-irrigator-aspirator (RIA) [99] or HARVEST ^®^ [100] designed to harvest stem cells for intraoperative surface coating of bone substitutes. However, an additional surgical approach or device material is required. During bone and joint surgery large amounts of mesenchymal stromal cells (MSCs) are released at the implantation site [101].

**Figure 2:**
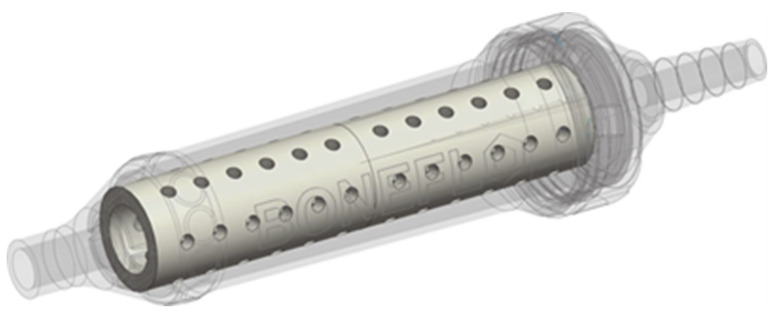
Illustrates a CAD-model of a suction handle for intraoperative stromal cell enrichment filled with a ceramic scaffold (©EFRE-Projekt Boneflo, Department of Orthopaedics and Trauma Surgery University of Duisburg-Essen, Germany).

Based on this principle, our research group investigated if osteogenic mesenchymal stem cells (MSCs) and osteoinductive cytokines can be enriched in a vacuum suction handle combined with bone substitute materials (ß-TCP and Allograft). The design of such a suction handle is shown in Figure 2.

**Figure 3:**
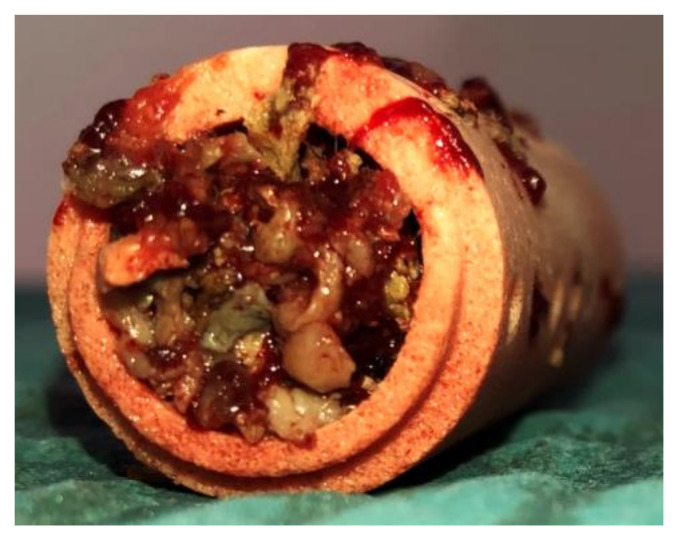
Shows a ß-TCP ceramic scaffold after intraoperative augmentation with cell-tissue composite (EFRE-Projekt Boneflo, Department of Orthopaedics and Trauma Surgery University of Duisburg-Essen, Germany).

On both bone substitute materials, high concentrations of mononuclear cells (MNC) could be found (Allograft: 1.94 ± 1.32 × 1010 vs. ß-TCP: 1.26 ± 1.03 × 1010, *p* = 0.145). The harvested cell-tissue composite on a β-TCP ceramic scaffold after intraoperative augmentation is visualized in Figure 3.

The cytochemical staining demonstrated high osteogenity of the cell-tissue composite (CTC). In total, 104 human cytokines, chemokines, growth factors, hormones and other soluble proteins were detected in the cell tissue composite from the surgical vacuum filter [102]. 

## 8. Conclusions

Functionalization of bone substitute materials is complex and the development has not yet been completed. The two main goals of creating functional biomaterials are prevention of infection and enhancement of bone healing. The addition of antibiotics to bone substitutes is considered technically difficult and the cytotoxic side effects must not be underestimated. The supplement with osteoinductive substances (e.g., rhBMP-2) lead to accelerated and improved bone healing. However, erratic bone formation is still a serious complication, especially in spine surgery. Future studies should focus on the resistogram-guided, patient-specific loading with antibiotics to treat IRI sufficiently.
